# The Founder of the Red Cross Association

**Published:** 1910-11-05

**Authors:** 


### The Founder of the Red Cross Association.

Not long after the death of Miss Nightingale follows that of one who may with justice be regarded as among the foremost of her disciples; on October 27 passed away at Heiden, Switzerland, Henri Dunant, founder of the International Red Cross Association and Nobel prize-winner, in his eighty-third year. From youth onwards this unassuming philanthropist interested himself constantly in championing the cause of the oppressed, wherever they might be found. A fervid admirer of Mrs. Beecher Stowe, he was prominent sixty years ago as a denouncer of slavery, as it existed both in the East and in the West; but it was in 1859 that his lifework first began to claim his attention. More by accident than by design he happened to be present at the battle of Solferino, and to witness the ghastly sufferings of the wounded of both sides, on the field and afterwards in the hospitals. With the help of an improvised ambulance service, which he organised from among the local inhabitants, he did what he could for the wounded, indifferent to their nationality. "Dunant," says the *Times*, "converted churches, public buildings, and private houses into hospitals, nd did his utmost to lessen the sufferings of the victims."

The harrowing scenes which M. Dunant then witnessed bore fruit not long afterwards in his pamphlet entitled "Un Souvenir de Solférino," which was translated into many languages, and is a most eloquent exposition of those horrors for which he was even then considering his scheme of alleviation. The example of Miss Nightingale's Crimean work, then fresh in everyone's mind, convinced him that permanently organised volunteer workers, drilled and instructed, were a solution of the question far preferable to makeshift and inefficient, though well-intentioned, amateurs. On returning to Geneva he began to advocate such a body, and to insist as a vital necessity that it should be afforded protection against capture and be treated as neutral by belligerents. To carry through such a scheme, derided for a time as Utopian, without incurring the hostility of military authorities, was no easy task; but M. Dunant and the committee of five appointed at Geneva in 1863 laboured incessantly and strenuously to gain their objects, and in 1864 the International Diplomatic Convention met at Geneva and signed the first of those treaties which have done so much to mitigate the sufferings of the wounded in war-time.

With its usual disregard of its greatest benefactors, the world promptly forgot M. Dunant, who was not sufficiently a self-seeker to succeed financially amid the selfish jostle of modern competition. After losing the whole of his fortune by the failure of a financial enterprise, he lived for long in great poverty; there were times when—let the world blush to own it—he dined on a crust, chalked his collar, and blackened his clothes with ink. Eventually the straits to which this unselfish and benevolent creditor of mankind were reduced became known; a committee was formed to help him, and in 1901 the award of the Nobel Prize justly recognised his claims to honour and lifted him out of the mire of destitution. His three inspirers and exemplars were Miss Nightingale, Mrs. Fry, and Mrs. Stowe; and it is a distinction which not even that heroic trilogy would despise thus to have stimulated the energies and provoked the emulation of a man whose works have done as much for humanity as those of the late originator of the Red Cross movement.

